# Anti-Typhoid Inoculation

**Published:** 1917-06

**Authors:** 


### Anti-Typhoid Inoculation.

The Canadian Department of Militia and Defense has just announced that for the twelve months ending December 31, 1916, only 167 cases of typhoid fever were reported as having occurred amongst the many thousands of men of the Canadian Expeditionary Force in Canada, notwithstanding the fact that typhoid fever is endemic in all parts of Canada, and is a disease especially affecting young adults from 17 to 30 years of age. This comparative freedom on the part of the Canadian Expeditionary Force is seen to be most striking when it is recalled that during the Boer war one man out of every nine in the British forces in South Africa was invalided through this disease, and that in the Spanish-American war, of 107,000 men in the camps at Tampa, Florida, and elsewhere, who had not left the shores of the United States, 20,000 contracted the disease. The remarkable change can only be attributed to inoculation. The Provincial Board of

Health for Ontario has supplied to date all the typhoid and paratyphoid vaccine used by the entire Canadian Expeditionary Force (about 450,000 men). In all, nearly 600,000 doses have been supplied free of cost.—*British Medical Journal,* March 31, 1917.

---

The young physician was tired, but, as he settled back in his easy chair, and his newly wedded wife took a seat beside him, he asked affectionately:

"And has my little wife been lonesome?"

"Oh, no," she said animatedly; "at least, not very. I've found something to busy myself with."

"Indeed!" he said. "What is it?"

"Oh, I'm organizing a class. A lot of young girls and married women are in it, and we're teaching each other how to cook."

"What do you do with things you cook?"

"We send them to the neighbors."

"Dear little woman," said he, "always thoughtful of your husband's practice."

